# Multi-Input Deep Convolutional Neural Network Model for Short-Term Power Prediction of Photovoltaics

**DOI:** 10.1155/2022/9350169

**Published:** 2022-09-20

**Authors:** Huimin Zhang, Yang Zhao, Huifeng Kang, Erzhao Mei, Haimin Han

**Affiliations:** ^1^College of Mechanical and Electrical Engineering, Henan Technical Institute, Zhengzhou, Henan 450042, China; ^2^Collegeof Chemical Engineering, Henan Technical Institute, Zhengzhou, Henan 450042, China; ^3^College of Aeronautics and Astronautics, North China Institute of Aerospace Engineering, Langfang, HeBei 065000, China

## Abstract

Along with the increasing prominence of energy and environmental issues, solar energy has received more and more extensive attention from countries around the world, and the installed capacity of photovoltaic power generation, as one of the main forms of solar energy development, has developed rapidly. Solar energy is by far the largest available source of energy on Earth, the use of solar power photovoltaic system has the advantages of flexible installation, simple maintenance, environmentally friendly, etc., by the world's attention, especially the grid-connected photovoltaic power generation system has been rapid development. However, photovoltaic power generation itself is intermittent, affected by irradiance and other meteorological factors very drastically, and its own randomness and uncertainty are very large, and its grid connection affects the stability of the entire power grid. Therefore, the short-term prediction of photovoltaic power generation has important practical significance and guiding meaning. Multi-input deep convolutional neural networks belong to deep learning architectures, which use local connectivity, weight sharing, and subpolling operations, making it possible to reduce the number of weight parameters that need to be trained so that convolutional neural networks can perform well even with a large number of layers. In this paper, we propose a multi-input deep convolutional neural network model for PV short-term power prediction, which provides a short-term accurate prediction of PV power system output power, which is beneficial for the power system dispatching department to coordinate the cooperation between conventional power sources and PV power generation and reasonably adjust the dispatching plan, thus effectively mitigating the adverse effects of PV power system access on the power grid. Therefore, the accurate and reasonable prediction of PV power generation power is of great significance for the safe dispatch of power grid, maintaining the stable operation of power grid, and improving the utilization rate of PV power plants.

## 1. Introduction

Energy is the material basis for human survival and development and is the power source for economic construction and social development [1]. With the rapid development of human society, energy consumption is growing exponentially, and relying on the use of a single energy source must not be able to meet the demand for energy in human society [2]. Stand-alone photovoltaic systems are photovoltaic power generation systems that rely entirely on solar cells, and the output of the photovoltaic array is its only source of energy [3]. The solar photovoltaic principle is now one of the most common technical means of obtaining solar energy resources and shares the responsibility of reducing carbon dioxide emissions and reducing the greenhouse effect with other renewable energy technologies [4]. Due to the many influencing factors related to PV power output, the output characteristics are cyclical, fluctuating, and stochastic, while at the same time, the stability of the power system is definitely threatened by the large grid connection of PV plants [5]. Therefore, it is necessary to forecast the PV power generation in order to take corresponding countermeasures.

Photovoltaic power generation system is a form of power generation that directly converts solar energy into electrical energy using the photovoltaic cell's photovoltaic conversion principle. The composition mainly includes solar panels, solar controllers, battery banks, and inverters [6]. And once the weather changes during the day, the impact on the PV output power is also great, when there are clouds on a sunny day, the passing of a cloud can lead to significant fluctuations in the PV power output power [7]. That is because the PV power generation system power generation is affected by a variety of factors, its output has obvious periodicity, volatility, and randomness making it an uncontrollable source relative to the power system after grid-connected operation, and when the scale of PV power plants is large will inevitably have an impact on the safe and stable operation of the power grid [8]. PV power prediction is based on numerical weather forecast data or actual measured data and then combined with the geographic coordinates of the PV plant and specific geographical characteristics of the parameterization scheme, to establish a prediction model and algorithm to achieve the prediction of the output power of the PV plant in a certain period of time in the future [9].

The ultra-short-term prediction (<4 h) mainly adopts the mixed methods of statistics and physics. The main principle is to predict the cloud movement according to the satellite cloud images taken by geosynchronous satellites, predict the radiation intensity reaching the ground, and predict the power through the solar radiation intensity and power conversion efficiency model. It is generally used for photovoltaic power generation control, power quality evaluation and *R*&*D,* and design of photovoltaic power station components.

The short-term prediction (<48 h) is mainly based on NWP (weather forecast information) data. By establishing the mapping relationship between historical input data and historical output power, the predicted value of output power of photovoltaic power stations can be obtained. It is generally used for power balance and economic dispatch of power system, formulation of day ahead generation plan, power market transaction, transient stability assessment, etc.

Medium and long-term prediction (>1 week) is mainly used for system maintenance arrangement, power generation prediction, etc.

Accurate PV power prediction can not only provide a reliable basis for power grid generation planning, peak and frequency regulation, tide optimization, equipment maintenance, and other scheduling decision-making behaviors. Moreover, it can provide technical support for multienergy complementary and coordinated control of wind, light, water, and fire storage, and is one of the key technologies to provide the ability of the grid to accept intermittent power at scale [10]. Multi-input deep convolutional neural networks have received high attention for their high algorithmic performance when they were proposed, and especially since the rise of deep learning, convolutional neural networks have once again attracted a lot of attention from researchers [11]. Accurate prediction of photovoltaic power output helps the power department to adjust the dispatching plan in a timely manner, coordinate the coordination of traditional power sources and photovoltaic power generation, meet the balance of power supply and demand, and ensure the reliable operation of the power grid [12]. Multi-input deep convolutional neural networks can use different convolutional kernels to extract different feature information of images, and its unique mechanism of local perceptual field and weight sharing can greatly reduce its network parameters and accelerate the training efficiency of the network [13]. The multi-input deep convolutional neural network model can help reduce the rotating backup capacity and operation cost of the power system, improve the economy of grid operation, and thus promote the faster and better development of grid-connected photovoltaic power generation.

The innovation points of this paper are given as follows:

1. A multi-input deep convolutional neural network model is used to optimize the BP neural network PV power prediction model, which is of great theoretical and practical significance for alleviating energy and environmental problems, improving the energy consumption structure, improving the performance of distributed power generation systems, and developing the PV power generation industry.
2. The background of PV power generation is introduced from the perspective of environmental energy, relevant policies, and future grid architecture; the concept and significance of PV power prediction are presented.
3. In general, convolutional neural networks are mostly trained with a single supervised training or unsupervised training. In this paper, we propose a multi-input deep convolutional neural network model to address this problem, which can achieve a higher accuracy rate.

This paper presents a multi-input deep convolution neural network model for photovoltaic short-term power prediction. The research is divided into four parts. The first part expounds on the necessity of forecasting photovoltaic power generation and the general countermeasure background. In the second part, the short-term power prediction method of photovoltaic power generation based on a multi-input deep convolution neural network is analyzed. The contents of data sets with similar dates are determined, and the short-term prediction steps of photovoltaic power generation are described. The third part analyzes the application of multi-input depth convolution neural network model in power generation prediction. Through gray correlation analysis, the performance of multi-input deep convolution neural network model is analyzed. Finally, the full text is summarized. According to the error analysis and evaluation, this paper can compare different prediction systems and prediction methods, so as to find a more suitable method to improve the prediction accuracy and algorithm efficiency and make better use of the prediction results to serve the actual production.

## 2. Short-Term Power Forecasting Method of Photovoltaic Power Generation Based on Multi-Input Deep Convolution Neural Network

### 2.1. Determining Data Set by Similar Days

**Figure 1:**
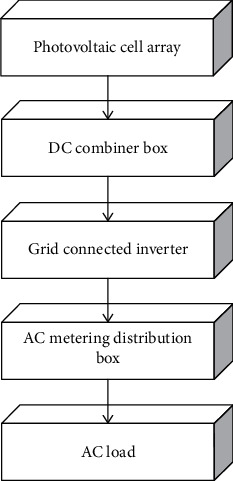
Block diagram of photovoltaic grid-connected power generation system.

Photovoltaic power prediction technology is a much-needed in-depth study, and accurate prediction of photovoltaic power generation has a better impact on the optimal scheduling of the power grid and power quality. It can be seen that the selection of similar daily data is very important and is an important topic for our future research [14]. Photovoltaic power generation systems include both stand-alone solar power generation systems and grid-connected solar power generation systems. The similar day has been one of the more commonly used concepts and methods in power load forecasting, wind speed forecasting, and photovoltaic power forecasting [15]. The photovoltaic power generation system component part mainly includes photovoltaic cell array, DC sink box, grid-connected inverter, AC metering distribution box, and AC load as shown in Figure 1.

First, daily data with similar weather types, i.e., similar influencing factors, to the day to be measured are selected as the training set of the model. These daily data are collectively called similar daily data, which are also historical daily data matched with the day to be tested. For an arbitrary signal *f*(*t*) or function that satisfies *f*(*t*) ∈ *L*_2_(*R*) and *ψ*(*t*) satisfies the wavelet tolerance condition, the continuous wavelet transform of *f*(*t*) is defined as follows:

(1) $$\begin{array}{c} {\text{WT}}_{f}\left(a,b\right)={\left|a\right|}^{-\left(1/2\right)}\int_{-\infty }^{+\infty }f\left(t\right)\psi \left(\frac{t-b}{a}\right)dt,a\neq 0\text{.} \end{array}$$

Due to the influence of external environmental and meteorological factors on PV power plants, such as PV array light angle, location, weather type, light intensity, temperature and humidity, wind speed, and cloud cover, PV power generation is prone to large fluctuations. Time-by-time solar radiation observation data constitutes a stochastic time series characteristic, which is highly random but still has deterministic laws. The half-sine model can portray this deterministic law and allocate the total daily radiation in each hour. PVG has three major characteristics: intermittency, periodicity, and randomness, together with inverter grid connection, islanding effect, and other factors, which affect the power quality of the grid and the reliability of the power supply. The large-scale access to large-capacity grid-connected photovoltaic power stations and distributed photovoltaic power sources has brought a series of problems to the planning, operation, and management of the power grid. At the same time, it is difficult to realize the complete scheduling of the power system for photovoltaic power generation. The incorporation of large-scale photovoltaic power stations into the power grid will certainly have an impact on the safe and reliable operation of the power grid. The model is simple, clear, and easy to implement, but the mechanical allocation ignores the stochastic nature of solar radiation. Meteorological and geographical factors affecting hourly solar radiation mainly include latitude: the lower the latitude, the stronger the solar radiation; weather conditions: there are more cloudy days and less solar radiation in Eastern China, Northwest China is deeply inland, with less precipitation, sunny days and more solar radiation; altitude: high altitude, thin air, good atmospheric transparency, and strong solar radiation; and sunshine duration: long sunshine duration and strong solar radiation. By discussing the meteorological and geographical factors affecting the hour-by-hour solar radiation, as well as the correlation analysis of the hour-by-hour solar radiation historical data, the input of the neural network-based hour-by-hour solar radiation prediction model was determined. Therefore, the prediction model of solar radiation is used to obtain the solar radiation values, after which the I/V electrical characteristics in PV modules are constructed under standard conditions. *ai*s the scale factor, reflecting the frequency information of the signal; *b* is the translation factor, reflecting the time information of the signal. The signal power spectrum of wavelet transform can be defined as follows:

(2) $$\begin{array}{c} S\left(\omega \right)=\frac{2\pi }{N}\frac{\sum_{i=1}^{N}{\left|W_{s}\left({a,\text{iT}}_{g}\right)\right|}^{2}}{\Delta \omega }. \end{array}$$

**Figure 2:**
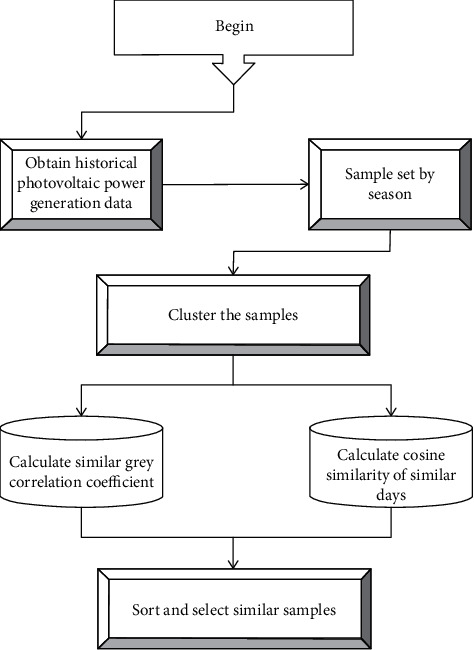
Principle of sample selection for similar days.

PV cell arrays are the most important part of a PV power generation system because only PV cells can convert sunlight into electricity. Generally, cell arrays can be arranged in two ways: in series and in parallel. Similar samples are selected according to the ranking of the integrated index values and used as input to the PV power prediction model. The principle of similar sample selection is shown in Figure 2.

Second, the PV power prediction is performed by statistical method or hybrid method, the core idea is to organize and analyze the data of historical power and historical influencing factors in order to discover the relationship between influencing factors and PV power output. The local spatial correlation between layers is used to connect the neuron node of each adjacent layer only with the upper layer neuron node, that is, close to it, local connection, and a multilayer forward network is built with the function to calculate the output *M* of the hidden layer:

(3) $$\begin{array}{c} M_{j}=f\left(\overset{n}{\underset{i=1}{\sum }}W_{ij}X_{i}-b_{j}\right),j=1,2,...,m\text{.} \end{array}$$

Historical power generation data can be obtained through the data acquisition system of the PV power system. These data, also known as the time series of power, have a high autocorrelation and already contain the influence of environmental factors such as light angle and location on the power series. In order to be able to meet the application in various weather type conditions, it is necessary to add meteorological weather forecast information to correct the predicted values. A chaotic optimization neural network prediction model was developed by unifying the historical data of hour-by-hour solar radiation based on the maximum sunshine hours of the year. The model is initialized with a filter of size *k* for the convolution operation, whose width is the same as the word vector dimension. The convolution operation is shown in the following equation:

(4) $$\begin{array}{c} c_{i}^{k}=\sigma \left(\sum \left(C\left[i,i+k\right]\circ H_{k}\right)+b\right), \end{array}$$

*σ*—Sigmoid activation function number. *C*[*i*, *i*+*k*]—Word vector sequence. *H*_*k*_*—*Convolution kernel

This is used as a basis to obtain the electrical characteristics around the variation of solar radiation intensity and temperature. After that, the short-term power values of the solar power system are obtained in relation to the MPPT conversion efficiency, etc. The controller serves as the data collection and monitoring of the PV system, and the main role of the controller is to monitor the PV system within a normal operating range to ensure that the system is always in the maximum efficiency working range.

Finally, using the similar day principle, the daily data with similar weather types, i.e., similar influencing factors, as the training set of the model are selected. Meanwhile, the PV power time series also has a certain periodicity, and the power value varies when it is in different seasons or different weather types, and the influencing factors such as light intensity, temperature, and humidity will cause fluctuations in the power time series. The Swish function is chosen as the activation function of the network model to improve the classification accuracy of the images. Its mathematical expression is given by the following equation:

(5) $$\begin{array}{c} f\left(x\right)=x\cdot \sigma \left(\beta x\right)\text{,}\\ \sigma \left(x\right)=\frac{1}{1+\exp\;\left(-x\right)}\text{,} \end{array}$$

*σ*(*x*), sigmoid activation function number.

The prediction results are analyzed for errors, and it is concluded that the chaotic optimization neural network prediction model can accurately reflect the time-by-time solar radiation change pattern. The general controller consists of two parts, the first one is the CPU because the collected and monitored data need to be processed by some analysis in order to enable the staff to better monitor the system operation status; the second part is the AD converter, whose main role is to collect the PV system operation data in real-time with high accuracy requirements. Parameters such as conversion efficiency and array area have been implicitly included in the historical power generation data because in the historical power generation time series of the array, all the power generation time series come from the same power generation system. The data itself contains the system information of the PV array, but variations in solar irradiation intensity and atmospheric temperature must be considered in the selection of input variables.

### 2.2. Short-Term Forecasting Steps of Photovoltaic Power Generation

**Figure 3:**
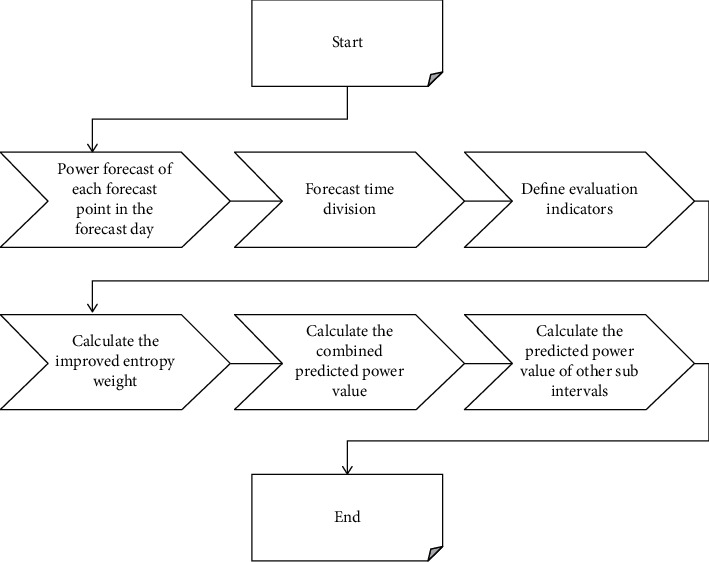
Short-term forecasting steps of photovoltaic power generation.

When a PV power generation system is connected to the grid, it is an uncontrollable source relative to the power system. When its proportion in the system is small, these generation characteristics do not have a significant adverse effect on the safe operation of the grid. Therefore, there is a need for a method that can simulate the distribution characteristics of prediction errors without any preconditions. The prediction of PV power generation portfolio is shown in Figure 3.

First, the formula for calculating the total output power of the PV power system is determined based on the composition characteristics of the PV arrays that make up the PV power system. The radial basis function neural network prediction model is established by taking the extra-atmospheric radiation, atmospheric quality, image brightness, and cloudiness as input factors and the surface radiation as output. To use multi-input deep neural network for PV power prediction, the number of nodes in the three layers of input, implicit, and output layers and the transfer function between the layers need to be determined, and then the prediction model is constructed. It is similar to the traditional neural network model. The input is added to the network and the output is calculated for each input vector and the value of each correlation weight *W*_*ji*_ is corrected according to the following equation:

(6) $$\begin{array}{c} W_{ji}\left(t+1\right)=W_{ji}\left(t\right)+\Delta W_{ji}\left(t\right). \end{array}$$

The indirect prediction method based on solar radiation intensity is to first use the data of solar radiation to construct a relevant model to predict the intensity of solar radiation. The number of nodes in the input layer mainly depends on the input variables including PV power history data and meteorological data, where the meteorological data are mainly daily light intensity, temperature, relative humidity, wind speed. After that, the physical principle of solar cells and photoelectric conversion efficiency is used as the basis to construct the empirical expression formula of the relationship between solar radiation intensity and photoelectric conversion efficiency as well as MPPT conversion efficiency and other mutual relationships. The conversion function is given as follows:

(7) $$\begin{array}{c} x^{\prime }=\frac{2\left[x-0.5\left(\max\;+\min\right)\right]}{\max\;-\min}, \end{array}$$

max—The maximum value of the sample. min—Minimum value of sample data. *x*—The value of the current sample point. *x*′—Normalize the calculated value.

Secondly, according to the solar radiation prediction model, the instantaneous solar radiation intensity value of the corresponding time interval on the prediction day is predicted as the input information for predicting the maximum theoretical total output power of the PV array or the maximum theoretical output power of the solar cells alone. Learning rate is a key parameter for short-term prediction of PV power generation, representing the step length of each step, which is important for the training of the model and symbolizes the model. It is important for the training of the model and symbolizes the generalization ability of the model, but if the generalization level is too high, the model prediction accuracy will be reduced due to overfitting. In order to make the input data smooth and prevent pseudoregression, preprocessing is performed on the data, most commonly wavelet analysis, empirical modal decomposition, etc. The irradiation intensity affects the output efficiency of the photovoltaic effect from time to time, because the main element in the photovoltaic cell material is silicon, with the light radiation intensity, the electric field can be generated within the silicon material cell, due to the electric field in the external presence of load access, and then generate current and output electric power. When the learning rate exceeds a certain range, the step size is too large, the model training accuracy is not high, or is too small, the running speed is slow, the CPU memory consumption is high and the model complexity becomes high, the generalization ability becomes poor. For a given input, *x* ∈ *R*^*d*^, max out hidden layer implements the function given by the following equation:

(8) $$\begin{array}{c} h_{i}\left(x\right)=\max\left(z_{ij}\right),j\in \left[i,k\right]\text{,}\\ z_{ij}=x_{T}W..{._{i}}_{j}+b_{ij}\text{.} \end{array}$$

Finally, according to whether the maximum theoretical output of the PV array is predicted, or the maximum theoretical output of the solar cells alone, the comprehensive efficiency of the PV array is calculated, and the short-term prediction of the PV power generation system output is based on solar radiation and PV power generation system model is obtained. If the external meteorological conditions, constant temperature and pressure, and irradiance change within a certain interval, it will bring little effect on the open circuit voltage of PV panels, in addition, the relationship between light intensity and PV power output power shows a positive correlation. Due to the diurnal fluctuation of PV power generation, the data collected from PV plants at night has little relationship with the actual PV output, so in order to maintain the consistency of the data, it is necessary to establish a uniform time window range for historical data, which plays an important role in PV power prediction modeling. Meteorological data of different regions and properties are continuously input into the computer, and through a certain prediction model, it is coordinated in terms of power and heat. The initial field, in which the mass field and the light field are basically in balance, is obtained and provided to the prediction model. The four-dimensional assimilation is mainly composed of three parts: one is the prediction model; second is objective analysis; and third is initialization. The function of the model is to extrapolate the previous data to the current analysis time; analysis is to combine the information of model prediction with the current observation data and interpolate it into the grid; and initialization is to filter the high-frequency gravity wave in the analysis field to ensure the stability of calculation. The correction and update of weights and thresholds of multi-input deep convolutional neural network also come from the negative gradient descent method, so once the corresponding nondifference function in the neural network becomes 0, that is, to say, the gradient becomes zero at this point, then the power threshold will not be updated to a better value, i.e., the update will stop. However, as the installed capacity of grid-connected photovoltaic power generation continues to expand, its proportion in the grid increases year by year, and its power generation volatility will cause an impact on the power system, directly affecting the safe and stable operation of the power system.

## 3. Application Analysis of Multi-Input Depth Convolution Neural Network Model in Power Generation Forecasting

### 3.1. Grey Correlation Analysis

Based on a multi-input deep neural network-based output power prediction model for photovoltaic power generation systems, a method to improve the accuracy of power prediction using gray correlation is proposed in order to reduce the volatility of power generation and the impact of randomness on power systems. Gray correlation analysis measures the degree of correlation between two variables by analyzing the relationship between them to express the degree of influence of one quantity on the other.

**Figure 4:**
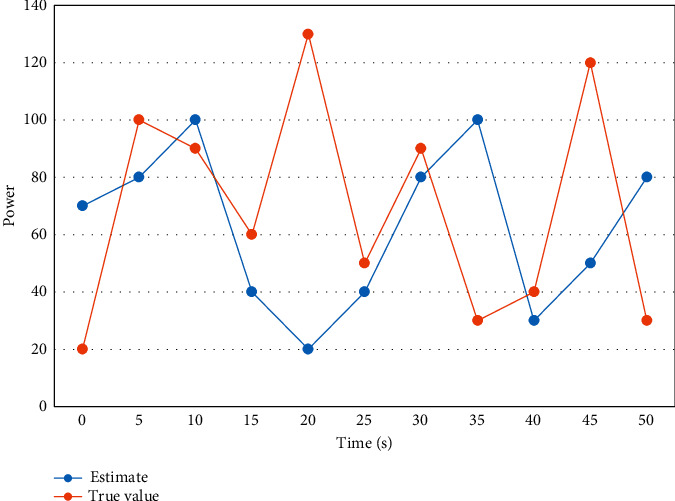
Comparison curve between real value and predicted value of power series on nonsunny days.

**Figure 5:**
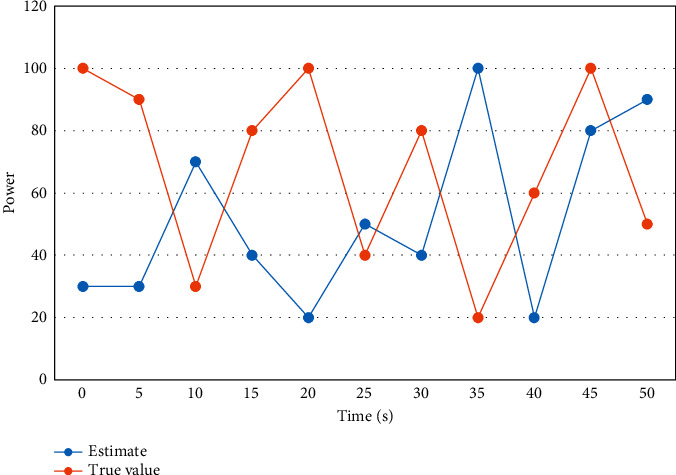
Comparison curve between real value and predicted value of power series on sunny days.

First, all data are normalized and the absolute value of the difference is calculated for each sample point. Using weight sharing makes neurons sharing the same weights detect the same feature at different locations, and the neurons sharing the same weights are organized into a two-dimensional plane to obtain the feature map. The magnitude of PV output power and the trend of PV output power is more or less different for different seasons, day types, and temperatures. To better demonstrate the model's prediction effect for sunny and nonsunny day types, the comparison curves between the prediction results and the real values for sunny and nonsunny day types are shown in Figures 4 and 5.

The sample space is then mapped to a high-dimensional or even infinite-dimensional feature space by nonlinear mapping, making it possible to apply the linear learning machine method in the feature space to solve problems such as highly nonlinear classification and regression in the sample space. The nonlinearity test is effective in detecting the presence of nonlinear features in the time series and is employed by using the ADF test in EViews software. The results of the ADF unit root test for the time series of power and each influence factor are shown in Table 1.

**Figure 6:**
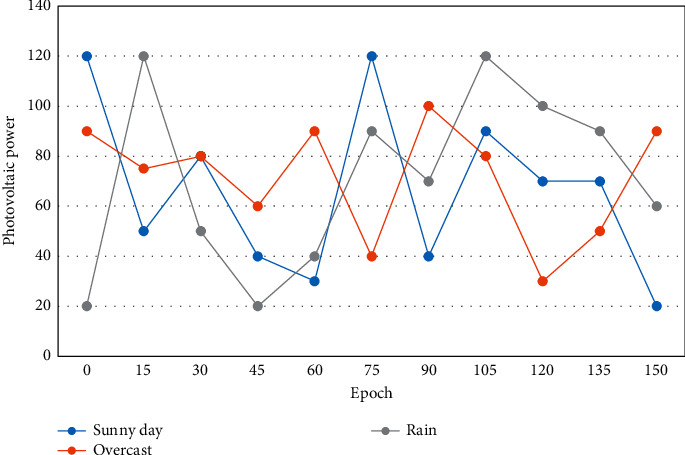
Photovoltaic power generation in different weather types.

Next, the maximum and minimum values of the sample points are found and the corresponding correlation coefficients of each are calculated. From the feature extraction point of view, local perceptual fields on the two-dimensional space can extract primary visual features from the two-dimensional images. For example, endpoints, corner points, and edges at specific angles. Subsequent layers can obtain higher-level, more abstract features by combining these primary features. The day type obtained from the meteorological department is only rough information, and when linked with PV output data, it does not necessarily represent the PV output under that weather condition accurately. The PV power curves under 3 typical weather types: sunny, cloudy, and rainy are shown in Figure 6.

In a convolutional layer, the parameters in the convolutional kernel are shared, i.e., the values of the parameters in a convolutional kernel remain the same during the convolution of all the image data. The process is that the error of the output signal is finally back-propagated from the hidden layer to the input layer according to the original pathway, and then the obtained error signal of each layer is assigned to all neuron units of each layer. Considering this situation, further filtering of the day types in the historical data series should be done by combining weather forecast data and the average PV output values under different day types in various seasons and subordinates, using the day type Euclidean distance. The process is to perform quantile regression calculation on the obtained prediction series and the corresponding error series after completing the point prediction to obtain the power prediction intervals at different quantile levels at a specific confidence level.

Finally, all the correlation coefficients in each column are averaged to obtain the result. The closer the result is to 1, the higher the degree of correlation. The multi-input deep convolutional neural network uses local connections and weight sharing to extract the local features of the input at each position of the input in a convolutional manner, which effectively simulates the simple cells in the primary visual cortex of primates. Each neuron changes the individual network connection weights based on this signal, which finally makes the error signal gradually decrease. Reducing the size of the feature mapping to a constant feature set not only regulates the complexity of the network but also helps to improve generalization by reducing overfitting. If the prediction accuracy is to be improved, the training error can be further reduced by increasing the number of hidden layers of the multi-input depth network. However, the structure of the neural network becomes more complex and the number of modal functions generated is larger, which brings the problem of a large model and long training time if all of them are input to the prediction network.

### 3.2. Performance Analysis of Multi-Input Depth Convolution Neural Network Model

**Figure 7:**
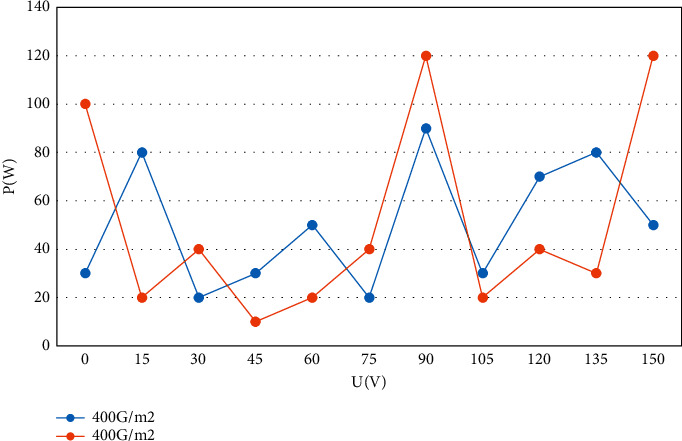
Power-voltage output curve under different light intensities.

A multi-input deep convolutional neural network model generally consists of a convolutional layer, a pooling layer, a fully connected layer, and an output layer. Its learning process is divided into two aspects: i.e., the forward transmission of the working signal and reverse transmission of the error signal. The performance analysis of the multi-input deep convolutional neural network model in power generation prediction has three steps, as shown in the following section:

First, the original image is converted into a feature map by convolution operation through a convolution layer, where the activation function adds a nonlinear factor to the feature map. In the wind data features, although the wind speed and wind direction are similar to the power correlation, the wind speed has a more direct impact on the PV panels. That is after the input has been extracted layer by layer to learn higher-level features, only the features obtained in the last stage are fed into the classifier, and the features at that level have the same scale of a perceptual field on the input image. Therefore, the data are preprocessed, and all the input features are normalized to remove the magnitudes for consistency analysis. Then, the gray correlation analysis is used to filter the features that are more correlated with the actual power output, which reduces the overall computational complexity and improves the accuracy of the model. Since the results of the training process do not match the expected values, the weights and thresholds are revised to keep approaching the expected output values. The distance between a sample and the center of each class is calculated based on the classes that have been classified, and the class with the smallest distance is the class to which the sample belongs. In fact, the magnitude of light intensity directly determines the magnitude of PV power, and light intensity and PV power show a positive correlation. The output curves of power voltage under different light intensities are shown in Figure 7.

Second, the pooling layer then pools these feature maps to learn more advanced feature information. The neurons in the same feature map of the pooling layer extract local features at different locations in the previous layer, while for a single neuron, the features extracted are local features at the same locations in several different feature maps in the previous layer. During daytime operation, the higher the wind speed the better the heat dissipation effect on the surface of the PV panel, and the lower the surface temperature of the PV panel, which is favorable to the PV output. So here, by proposing a similar day selection algorithm and a method to determine the training sample for the weather information of the predicted day, the selected similar day, training sample, and predicted day have the same weather type and season type. And the temperature is close, and the training process and the prediction process are more targeted to improve the accuracy of the model in predicting solar radiation intensity under nonsunny weather type conditions. By establishing the mathematical models of direct solar radiation intensity and scattered solar radiation intensity, and using the calculation formula proposed by ASHRAE, the solar radiation energy at any time and in any region can be obtained. In addition, the cloud cover correction coefficient is obtained through the influence of clouds on the solar radiation intensity, which makes it possible to calculate the solar radiation intensity in nonsunny days. Comparing the predetermined accuracy with the errors present in the calculation, the procedure is completed when the result is higher than the predetermined accuracy or the number of learning exceeds the maximum number previously set. In order to take advantage of the decomposition of the deep convolutional neural network model and to reduce or avoid multiple random errors, the frequency sequences obtained from the decomposition of the model are reconstructed using the permutation entropy algorithm.  Table 2 shows the decomposition results of power and time series of each influencing factor in the nonsunny day training set.

**Figure 8:**
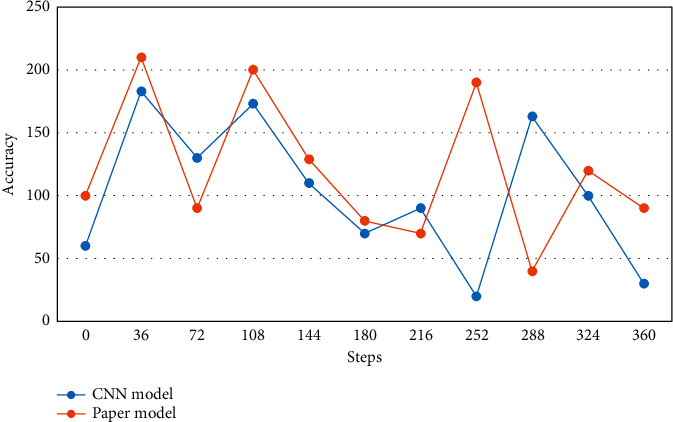
Comparison of accuracy (%) on CIFAR-10 data set.

Finally, the final output is passed through a fully connected layer, which in turn classifies the image with a classifier. When a support vector machine is used as the classifier with a supervised training method, the feature extraction phase and the training of the classifier with differentiable weights are allowed first, and then the features obtained in the feature extraction phase are kept constant for each weight, and the features obtained in the feature extraction phase are used to train the support vector machine. To more clearly compare the classification effects of the multi-input deep convolutional neural network model and the CNN model, Figure 8 shows the accuracy variation curves of this paper's model and the CNN model with the number of training steps on the CIFAR-10 dataset.

The magnitude of the weights in the network structure does not change in any way during the whole transmission process. If the output signal does not match the desired output signal during transmission, the process of error signal transmission occurs. The sparse features of the image are extracted by mapping the image to a lower dimensional space through multiple convolutional transforms and downsampling. The maximum and minimum temperatures of the predicted day are then queried, and the temperature Euclidean distance is calculated by combining the maximum and minimum temperatures corresponding to each day in history recorded in the day type library under the corresponding seasonal library in the weather forecast. Then the samples of the test set are classified and discriminated by selecting appropriate discriminant rules. The method avoids information overlap by studying the internal structure of the correlation matrix of the original variables and replacing multiple correlated variables with a few variables that are independent of each other. And these few independent variables retain the main information of the original variables, which can reduce the input dimension of the model and improve the model training speed.

## 4. Conclusions

This paper studies the short-term power prediction method of photovoltaic power generation, and proposes a multi-input deep convolution neural network model. This method solves the problem that the historical data of photovoltaic power generation after division is discontinuous in the time dimension. Using the daily type and temperature information released by the meteorological bureau, select the most relevant similar day, and then study the short-term prediction steps of photovoltaic power generation. It solves some error problems in the prediction process and improves the accuracy and stability of prediction. Finally, the application of multi-input depth convolution neural network model in power generation prediction is analyzed. And because the previous day is not always used as a similar daily input model, the error is large, so it is practical. It has important theoretical and practical significance for alleviating energy and environmental problems, improving energy consumption structure, improving the performance of distributed generation systems, and developing the photovoltaic power generation industry. At the same time, according to the error analysis and evaluation, different prediction systems and prediction methods can be compared, so as to find a more suitable method to improve the prediction accuracy and algorithm efficiency and make better use of the prediction results to serve the actual production.

## Figures and Tables

**Table 1:** ADF unit root test of power and time series of influencing factors.

	Power	Irradiance	Humidity
K-statistics	−7.25	−5.77	−18.2
Probability	0.0003	0.0002	0.0007
Smoothness	10%	20%	17%

**Table 2:** Ranking entropy results of time series decomposition results of concentrated power and various influencing factors in nonsunny training.

	IMF1	IMF2	IMF3	IMF4
Frequency	2.34	1.98	1.55	0.97
Power	1.56	1.27	1.63	1.28
Humidity	1.45	1.25	0.76	0.48
Ambient temperature	1.55	1.72	1.69	2.01

## Data Availability

The data used to support the findings of this study are available from the corresponding author upon request.
